# Stabilization of the Distal Radioulnar Joint with or without Triangular Fibrocartilage Complex Tear by an External Wrist Band Brace: A Cadaveric Study

**DOI:** 10.3390/healthcare10050828

**Published:** 2022-04-30

**Authors:** Seung-Han Shin, Taeyong Park, Eunah Hong, Dai-Soon Kwak, Yang-Guk Chung

**Affiliations:** 1 Department of Orthopedic Surgery, Seoul St. Mary’s Hospital, College of Medicine, The Catholic University of Korea, Seoul 06591, Korea; tumorshin@gmail.com (S.-H.S.); ygchung@catholic.ac.kr (Y.-G.C.); 2 Yonseihun Hospital, Gwangmyeong-si 14236, Korea; ddyyong@naver.com; 3 Department of Anatomy/Catholic Institute for Applied Anatomy, College of Medicine, The Catholic University of Korea, Seoul 06591, Korea; 2341568@naver.com

**Keywords:** wrist, external band brace, distal radioulnar joint, instability, triangular fibrocartilage complex tear

## Abstract

The purpose of this study was to investigate whether a watch-shaped external wrist band brace improves distal radioulnar joint (DRUJ) stability. Seven fresh cadaveric arms were used. Using a customized testing system, volar and dorsal translation forces were applied to the radius externally while the ulna was fixed. The test was performed with the forearm in neutral, 60° pronated, and 60° supinated positions, once without the brace and once with the brace applied. In each condition, the amount of translation was measured. Then, the triangular fibrocartilage complex (TFCC) was detached from the ulnar styloid process and the fovea ulnaris, and the same tests were performed again. Detachment of the TFCC significantly increased volar and dorsal translations in all forearm rotations compared to the intact condition (*p* < 0.05), except for the pronated dorsal translation of the radius (*p* = 0.091). Brace application significantly reduced volar and dorsal translations in all forearm rotations both in intact specimens and in TFCC-detached specimens (*p* < 0.05), except for pronated volar and dorsal translations in TFCC-detached specimens (*p* = 0.101 and *p* = 0.131, respectively). With the brace applied, the TFCC-detached specimens showed no significant difference in volar or dorsal translation in all forearm rotations compared to the intact specimens (*p* > 0.05). The external wrist band brace improved DRUJ stability in both normal and TFCC-torn wrists and reduced the DRUJ instability caused by TFCC tear to a near-normal level.

## 1. Introduction

The distal radioulnar joint (DRUJ) allows forearm pronation and supination. Because of the different radii of curvature of the sigmoid notch and the ulnar head, the motion in the DRUJ is composed of rotation and translation [1]. Motion in the DRUJ is limited to a physiologic range because of DRUJ stabilizers including bony conformation and soft tissues: the triangular fibrocartilage complex (TFCC) including dorsal and palmar radioulnar ligaments, the interosseous membrane, and the musculotendinous units, primarily the extensor carpi ulnaris and the pronator quadratus [1]. Among soft tissue components, ulnar insertion of the TFCC, especially foveal insertion, is considered most important for DRUJ stability [2,3].

DRUJ instability can cause ulnar-sided wrist pain with exacerbation during forced forearm rotation or axial loading, and the symptoms can be debilitating [4,5]. Such instability can be caused by fractures or soft tissue injuries including TFCC tears, which are generally indicated for surgery when accompanying DRUJ instability [6]. However, controversy remains, in that recent studies reported no long-term advantages of TFCC repair over conservative treatment in acute DRUJ instability following a distal radius fracture [7,8], and nonsurgical initial treatment is recommended for TFCC tears when the DRUJ is stable [9].

Conservative treatments of DRUJ instability and TFCC tears involve the immobilization of the DRUJ. The immobilization usually involves splinting or casting for weeks, but these treatments have limitations of restriction of activities and inconsistent outcomes [10,11,12,13]. Functional bracing is an alternative for limiting the motion in the DRUJ, with good outcomes reported even in chronic cases or after failure of other conservative treatments [14,15,16]. However, the number of such studies is very small, and the braces reported were bulky or had no biomechanical basis. The purpose of the present study was to investigate whether a watch-shaped external wrist band brace improves DRUJ stability, especially in the setting of TFCC tear.

## 2. Materials and Methods

### 2.1. Specimens

This study was conducted in compliance with the Act on Dissection and Preservation of Corpses of the Republic of Korea (act number: 14885) and was approved by the Institutional cadaver research committee of the College of Medicine, Catholic University of Korea (No. R19-A027). Written informed consent for use of the cadaver and consent for use of future research on the related materials were provided by all donors or authorized representatives. Seven fresh cadaveric arm specimens from four donors were disarticulated at the shoulder and enrolled in this study. The mean age of the specimens was 83 ± 9 years. Five specimen donors were female, and two were male. Four specimens were left arms, and three were right arms. No specimen had any skeletal deformity or soft tissue defect.

### 2.2. Concept of the Brace

**Figure 1:**
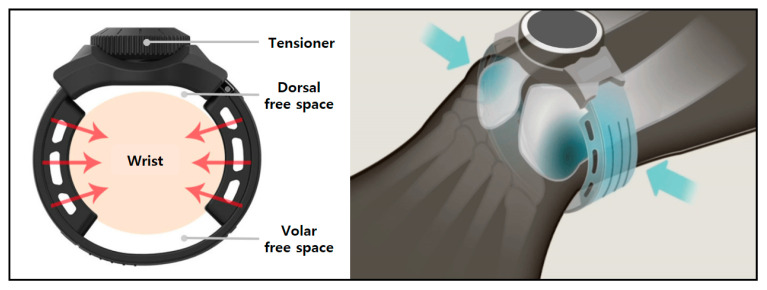
Structure and concept of the external wrist band brace.

The brace applied in this study is watch-shaped and worn externally just proximal to the ulnar head (Figure 1). The brace is designed to act as a tension band with more force applied in the radio–ulnar direction. Volar and dorsal free spaces of the brace enable this force distribution and also ensure blood flow return. The tension of the band is controlled stepwise by a rotary tensioner. In practice, the tension is adjusted by the user to a level producing adequate compression without pain. This self-adjustment of the brace tension allows long-term application of the brace, as in previous reports on DRUJ functional bracing [14,15,16]. However, in this experimental study, the applied tension was constant, at 0.8 kgf·cm torque of the tensioner dial.

### 2.3. Biomechanical Testing

**Figure 2:**
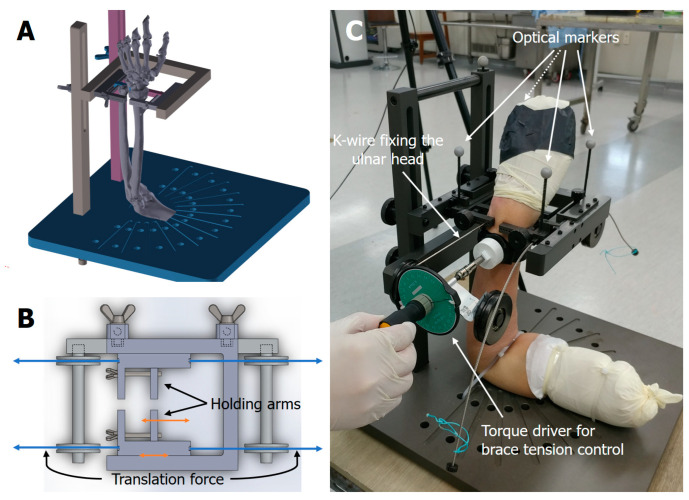
Customized testing system. (**A**) Scheme in 3D of the testing jig. The jig was designed to simulate a DRUJ ballottement test. (**B**) Structure of the holding tool arms. The translation force on the radius was applied through the string attached to the sliding part of the holding tool arms. (**C**) Actual test setting. The ulna was fixed with a K-wire, and the DRUJ translation was measured by detecting the motion of the radial optical markers in a volar–dorsal direction. The external wrist band brace was tensioned uniformly with a torque driver.

In this study, the amount of DRUJ translation was measured in cadaveric arms with intact soft tissue, in the same way as in the DRUJ ballottement test performed in clinical practice [17]. A customized testing system (Figure 2) was designed by the authors (SHS and DSK) referring to the in vivo DRUJ translation measurement device of Pickering et al. [18] It was fabricated using aluminum 6061 material and was anodized to prevent corrosion and improve surface hardness. Using the system, volar and dorsal translation forces of 20 N were applied externally to the radius while the ulna was fixed. The test was performed with the forearm in neutral, 60° pronated, and 60° supinated positions, first without the brace and then with the brace applied. In each condition, five cycles of 20 N preloading for 5 s were performed before measurement, and translation was measured three times. Then, the triangular fibrocartilage complex (TFCC) was detached from the ulnar styloid process and the fovea ulnaris through a transverse incision, and the same tests were performed. Comparisons were conducted between conditions for the same specimen.

### 2.4. Test Setting and Translation Measurement

The customized testing jig was designed to simulate a DRUJ ballottement test (Figure 2). The jig had two sets of sliding arms to hold the radius and the ulna externally. The sliding jig arms had holes for K-wires, so that the distal ulna could be secured to the holding arms of the jig. With the distal ulna fixed, the distal radius was moved volarly or dorsally by sliding the holding jig arms.

The specimen was set on the jig with the elbow flexed at 90°. The wrist was placed within the holding arms of the jig, and the upper arm was secured onto the base plate with three ropes so that the forearm could be in neutral, 60° pronation, or 60° supination positions according to the testing conditions. The distal ulna was secured to the holding jig arms with a K-wire placed through the holding arms and the ulnar head. The holding jig arms for the radius and the ulna were screw-tightened to hold each bone externally, and the holding jig arm for the ulna was fixed to the post of the jig. With the ulna fixed, a translation force was applied to the radius through the string wire attached to the sliding holding jig arm for the radius. The distance of the movement of the radial holding jig arm in the volar–dorsal direction was measured as the amount of DRUJ translation. For the brace-applied test conditions, the external wrist band brace was applied at the level just proximal to the ulnar head. The tension of the band brace was set uniformly at 0.8 kgf·cm torque of the tensioner dial.

To measure the amount of DRUJ translation, the researchers used a motion capture system and optical markers (Cortex 7.0, Motion Analysis Corp., Rohnert Park, CA, USA). Four optical markers were attached to the sliding part of the holding arms of the jig, two to the ulnar side, and two to the radial side. Three motion capture cameras (Kestrel 1300, Motion Analysis Corp.) were set to measure the movement of the radial optical markers relative to the ulnar optical markers. To calibrate the motion capture system, an L-frame and a 200 mm wand were used. After calibration, the wand length measured 199.99~200.02 mm repeatedly. Accuracy during continuous movement was verified by measuring the distance between two optical markers on the ulnar side. For 60 s at 60 Hz capture, the RMSE (root-mean-square error) was 0.012 mm.

### 2.5. Statistical Analysis

Statistical analyses were performed with IBM SPSS Statistics software Version 24.0 (IBM Corp., Armonk, NY, USA). The data were verified for normal distribution using the Shapiro–Wilk test. The significance of differences in the mean values of each measured parameter was analyzed by the paired *t*-test at a level of *p* < 0.05.

## 3. Results

**Figure 3:**
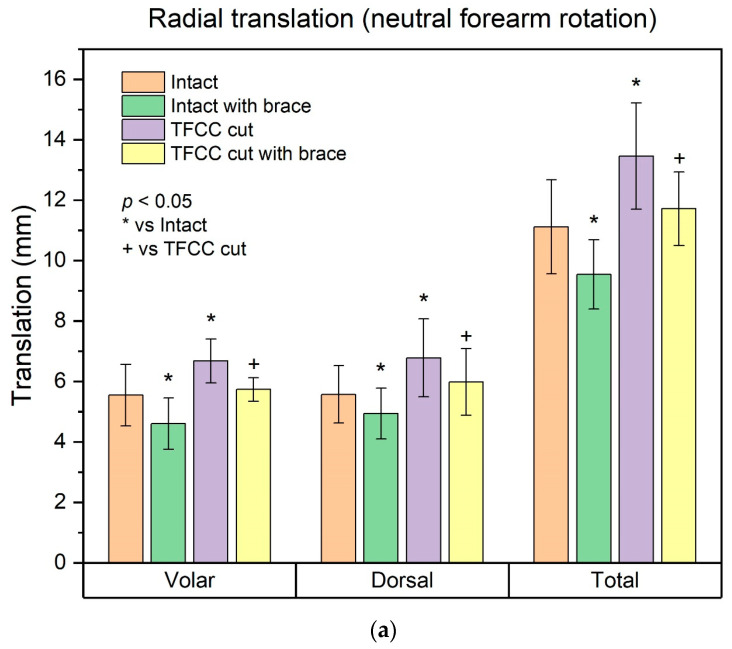

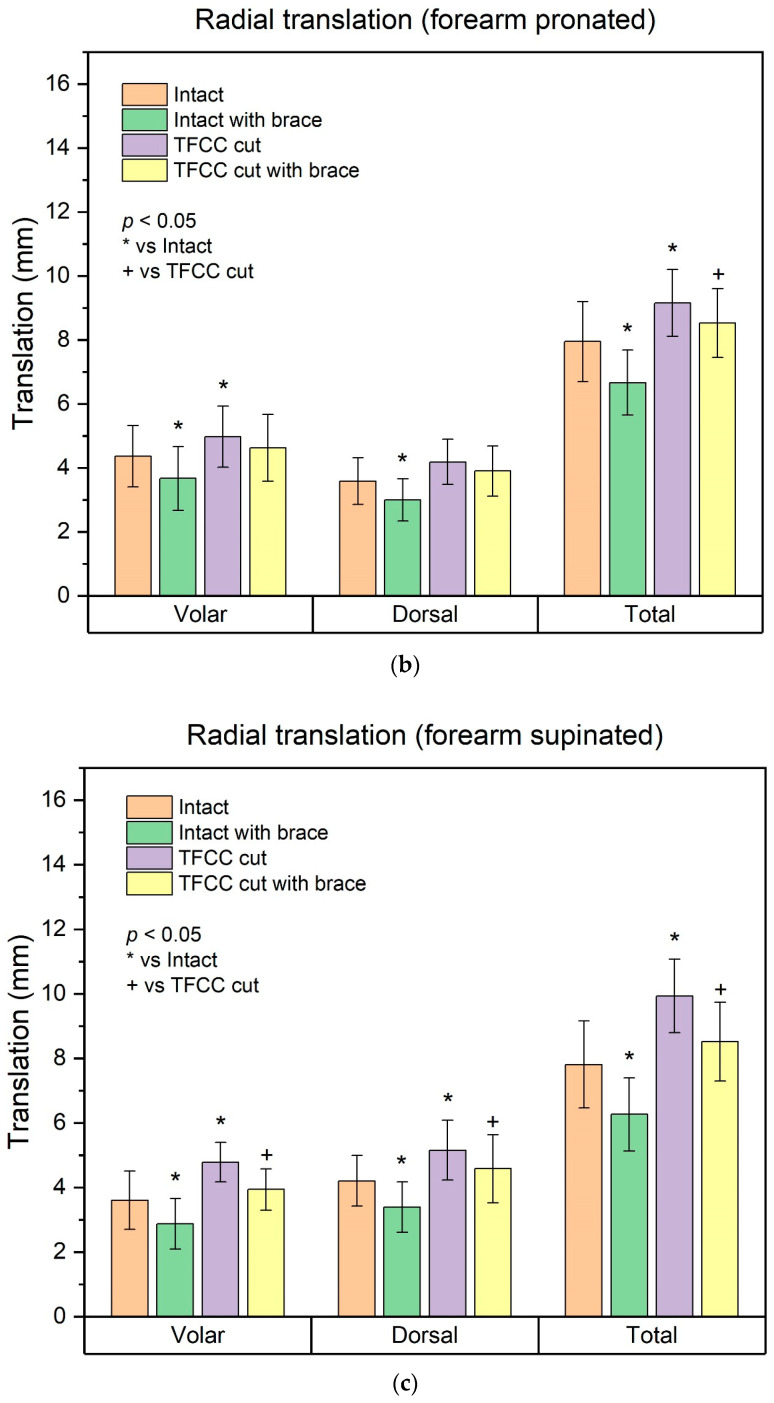
The amount of DRUJ translation in each condition. (**a**) Radial translation (neutral forearm rotation); (**b**) Radial translation (forearm pronated). (**c**) Radial translation (forearm supinated).

The results are summarized in Figure 3 and Table S1. TFCC detachment significantly increased volar and dorsal translations of the radius in all forearm rotations compared to the intact condition (*p* < 0.05), except for the pronated dorsal translation of the radius (*p* = 0.091). Brace application significantly reduced volar and dorsal translations of the radius in all forearm rotations both in intact specimens and in TFCC-detached specimens (*p* < 0.05), except for pronated volar and dorsal translations of the radius in TFCC-detached specimens (*p* = 0.101 and *p* = 0.131, respectively). With the brace applied, the TFCC-detached specimens showed no significant difference in volar or dorsal translation in any forearm rotation compared to intact specimens (*p* > 0.05).

## 4. Discussion

The results of this study demonstrated that an external wrist band brace improved DRUJ stability in cadaveric specimens with intact soft tissue. The brace reduced the amount of translation in both intact and TFCC-cut specimens. Especially, the brace restored the TFCC detachment-induced DRUJ instability to a level that was not significantly different from that of the intact condition.

The TFCC, including volar and dorsal distal radioulnar ligaments, is the most important stabilizer of the DRUJ [2,3]. Accordingly, in this study, detachment of the ulnar insertion of the TFCC (from both the ulnar styloid and the fovea ulnaris) resulted in significant DRUJ instability. The lack of statistical significance of the increase in pronated dorsal translation of the radius in the TFCC-cut condition can be attributed to secondary stabilizers of the DRUJ, such as the interosseous membrane [19]. However, despite the lack of statistical significance, there was some increase in pronated dorsal translation after TFCC detachment; a complete ulnar-side tear of the TFCC results in global instability of the DRUJ.

The result that the external wrist band brace restored the TFCC detachment-induced DRUJ instability to a near-normal level is the most impressive finding of this study. As mentioned in the introduction, controversy remains about the conservative treatment of TFCC tears. There have been reports on good outcomes of conservative treatments for TFCC tears with stable DRUJ [20] or those accompanied by distal radius fracture [7,8]. However, although initial conservative treatment is recommended for TFCC tears [21], those with DRUJ instability are generally indicated for surgery, and there have been few reports on their conservative treatment. Millard et al. reported the restoration of DRUJ stability with braces, but the braces were forearm-worn and bulky [16]. O’Brien and Thurn reported another type of orthosis for DRUJ instability, but the orthosis was bulky, and they did not objectively measure DRUJ translation [14]. Barlow reported a single case of a simple wrist band brace for TFCC tear, but it was not clear if the patient had DRUJ instability, and the DRUJ translation was not objectively measured [15]. In contrast, the current study objectively demonstrated restoration of TFCC detachment-induced DRUJ instability with a simple wrist-worn band brace. Considering the convenient watch-like structure and simple tension adjustment, this brace is an effective alternative for the conservative treatment of TFCC tears with DRUJ instability, when a sugar-tong splint or a cast cannot be applied.

The current brace also reduced DRUJ translation in intact specimens. The result suggest that the brace restricts the normal motion in the DRUJ. There has been a report that an external wrist band reduced both DRUJ translation and range of forearm rotation in a finite element model [22]. Although the range of motion was not evaluated in the current study, the reduced DRUJ translation of the intact specimen with the brace applied suggests that the brace also restricted forearm rotation. However, such a limiting effect of the brace might be helpful for preventing excessive translation or excessive rotation of the wrist that can result in a TFCC tear or other wrist stabilizer injuries. Future clinical studies on the preventive effect of the brace will be necessary to address this issue.

The current study has limitations. First, the test scheme simulating a DRUJ ballottement test can be affected by inconsistent pressure-compression of the soft tissue. However, the researchers tried to minimize errors using five pre-loadings and triplicated measurements and believe the test is more objective and consistent than the actual DRUJ ballottement test used in clinics. Second, the positions of the wrist and hand were not uniformly controlled. They were left in the natural position after setting the specimen on the customized jig. However, the position was the same in a single specimen, and the K-wire fixing the ulnar head and the holding arms of the jig were kept in the same position. Third, the tests were performed with a single brace tension and fixed forearm rotations. Further study is necessary to investigate the optimal brace tension, especially in clinical settings, and the effect of the brace on the range of forearm rotation.

In conclusion, an external wrist band brace improved DRUJ stability in both normal and TFCC-torn wrists and reduced the DRUJ instability caused by TFCC tear to a near-normal level. Considering the convenient watch-like structure and simple tension adjustment, the current brace can be an effective alternative for the conservative treatment of TFCC tears with DRUJ instability. The brace can also be considered for use on a normal wrist for protection against DRUJ injury caused by excessive translation.

## Data Availability

The data presented in this study are available on request from the corresponding author.

## Supplementary Materials

The following supporting information can be downloaded at: https://www.mdpi.com/article/10.3390/healthcare10050828/s1. Table S1. The results of DRUJ translation in each condition.
